# Forest Type, Bark Wounding, and Tapping: Their Combined Influence on Bacteria Biota of Styrax Paralleloneurus in Natural and Community Forest

**DOI:** 10.1111/1758-2229.70184

**Published:** 2025-09-04

**Authors:** Arida Susilowati, Margaretta Christita, Siti Halimah Larekeng, Adebola Azeez Lateef, Wenzi Ren, Abiodun A. Azeez, Rumella Simarmata, Yeni Khairina, Fiqriah Hanum Khumairah, Deni Elfiati, Fred O. Asiegbu

**Affiliations:** ^1^ Faculty of Forestry Universitas Sumatera Utara Deli Serdang Indonesia; ^2^ Research Center for Applied Microbiology National Research and Innovation Agency Cibinong Indonesia; ^3^ Faculty of Forestry Hasanuddin University Makassar Indonesia; ^4^ Department of Forest Science, Faculty of Agriculture and Forestry University of Helsinki Helsinki Finland; ^5^ Department of Plant Biology University of Ilorin Ilorin Nigeria

**Keywords:** bacteria, biodiversity, forests, next‐generation sequencing, *Styrax paralleloneurus*

## Abstract

*Styrax paralleloneurus* is a resin‐producing tree native to Sumatra, Indonesia. This study investigated the effects of tapping, bark wounding and forest type on bacterial biota in the stem of styrax in natural and community forests. Amplicon metagenomic sequencing of the 16S rRNA region was deployed to identify the bacterial communities associated with tapped and untapped trees across various environmental and experimental conditions. The results of the study showed that tapped trees had lower abundance and diversity of *Pseudomonas* compared to untapped trees, largely due to their increased exposure to external microbe communities and environmental elements. *Serratia* and *Pantoea* were more abundant in natural forest than community forest, while *Bradyrhizobium lablabi* was found abundantly in untapped trees. Additionally, the taxonomic analysis revealed distinct responses of bacterial genera to tapping and forest type, indicating that community forests could play a significant role in promoting biodiversity in forest ecosystems. This finding underscores the importance of community forests in biodiversity conservation. These insights can inform future conservation and management strategies to enhance biodiversity and underscore the need for sustainable forest management practices to maintain forest health and productivity.

## Graphical abstract

*Styrax paralleloneurus*, a resin‐producing tree from Sumatra, Indonesia and the diversity of bacterial communities within its stems, identified through amplicon metagenomic sequencing. It compares the bacterial abundance and diversity between forest types, bark wounding and tapping, highlighting the importance of community forests for biodiversity.

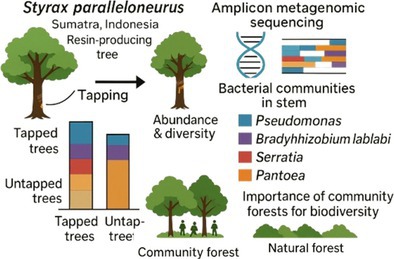

## 1 Introduction

*Syrax paralleloneurus* (previously *Styrax sumatrana*) is a frankincense‐producing plant native to Sumatra, Indonesia. The genus of Styrax is a major source of non‐timber forest products (Estiko 2020). Two main Styrax species have been reported to be a major source of benzoin resin, namely *Styrax paralleloneurus* Perkins and *Styrax benzoin* Dryand (Slamet et al. 2021). *Styrax paralleloneurus* is often known as Tobanese incense (Iswanto et al. 2021). Tobanese incense is a species of resin‐producing tree in the Styrax genus from the Indonesian province of North Sumatra in the districts of North Tapanuli, Humbang Hasundutan, Papak Bharat, Toba Samosir and Dairi. It is widely marketed because *S*. *paralleloneurus* resin has a wide range of applications, including cosmetics, fragrances, pharmaceuticals and traditional medicine (Sohail Akhtar and Alam 2022). Over the last few decades, community forest management with non‐timber forest products as the main commodity has not been supported by the proper yield regulation. The resin of Toba frankincense contains compounds that are used for industrial raw materials, medicines, cosmetics, natural insecticides and food and beverage preservatives. As a tree that produces high economic value non‐wood forest products, Styrax faces excessive exploitation. To harvest frankincense resin from the Styrax tree, farmers use the tapping method. Until now, tapping of incense resin has been done manually by wounding the trunk of the incense tree using a knife and bare hands. The activity of human touch certainly has various influences on the life of the Styrax tree, including changes in the microbiome diversity of the Styrax trunk. Moreover, tapping to obtain sap also induces wounds which trigger the plant's resistance to stress. Our hypothesis is that there are differences in microbial diversity in Styrax stems that have been tapped and those that have not. The preservation of biodiversity in community forests is a critical concern, as these ecosystems are often subject to varying degrees of human disturbance (Mir et al. 2022). Microbial communities associated with resin‐producing plants play pivotal roles in resin biosynthesis, degradation and overall plant health. In coniferous species such as *Pinus sylvestris* , resin‐rich environments harbour diverse microbial taxa, including bacteria like *Pseudomonas*, *Burkholderia*, *Azotobacter* and *Pantoea*, which are capable of tolerating and metabolising terpenes—the primary constituents of resin. Notably, certain *Pseudomonas* species have demonstrated the ability to degrade resin acids, utilising them as carbon sources (Martin et al. 1999; Vilanova et al. 2014; Ren et al. 2018; Terhonen et al. 2019).

In a forest ecosystem, various bacteria play a vital role in maintaining the health of trees, influencing their growth, and interacting with other organisms. *Paenibacillus polymyxa* is a diazotrophic bacterium that has been isolated from lodgepole pines ( *Pinus contorta* ) in British Columbia, Canada. This bacterium may provide benefits to its host by fixing nitrogen (Puri et al. 2020). Actinobacteria are recognised for their ability to decompose lignin and produce antibiotics that protect tree roots (Barka et al. 2016; Oyedoh et al. 2023). Meanwhile, *Brenneria goodwinii* and *Gibbsiella quercinecans* are linked to Acute Oak Decline, a disease that impacts oak trees (Brady et al. 2017). In tropical resin‐producing trees like *Boswellia sacra* , the source of frankincense, rhizospheric microbial communities—including endophytic fungi and bacteria—contribute to plant resilience under arid conditions and may influence resin quality and yield (Khan et al. 2017). Similarly, in *Dracaena cambodiana*, producer of dragon's blood resin, endophytic fungi such as *Cladosporium, Fusarium* and *Trichoderma* are implicated in resin formation processes, potentially by modulating flavonoid biosynthesis and glycosylation pathways (Li et al. 2025).

In agarwood‐producing species like *Gyrinops versteegii* , fungal inoculation—particularly with *Trichoderma* species—has been shown to enhance resin accumulation, suggesting a direct role of specific microbes in inducing resin biosynthesis (Mega et al. 2023; Mega and Kartini 2020). These findings underscore the significance of microbial partners in resin‐producing plants, highlighting their potential applications in sustainable resin production and plant health management.

Currently, research related to styrax mostly discusses productivity and cultivation, but no study has ever been conducted on the microbiota of this tree and the implications of human activity on its biodiversity. To better understand the impact of such disturbances, this study examines the phylogenetic relationships and microbial diversity of the medicinal tree species *Styrax paralleloneurus* in natural forests (free of human influence) and community forests with differing levels of human disturbance.

## 2 Experimental Procedures

### 2.1 Sample Collection and Field Study

**FIGURE 1:**
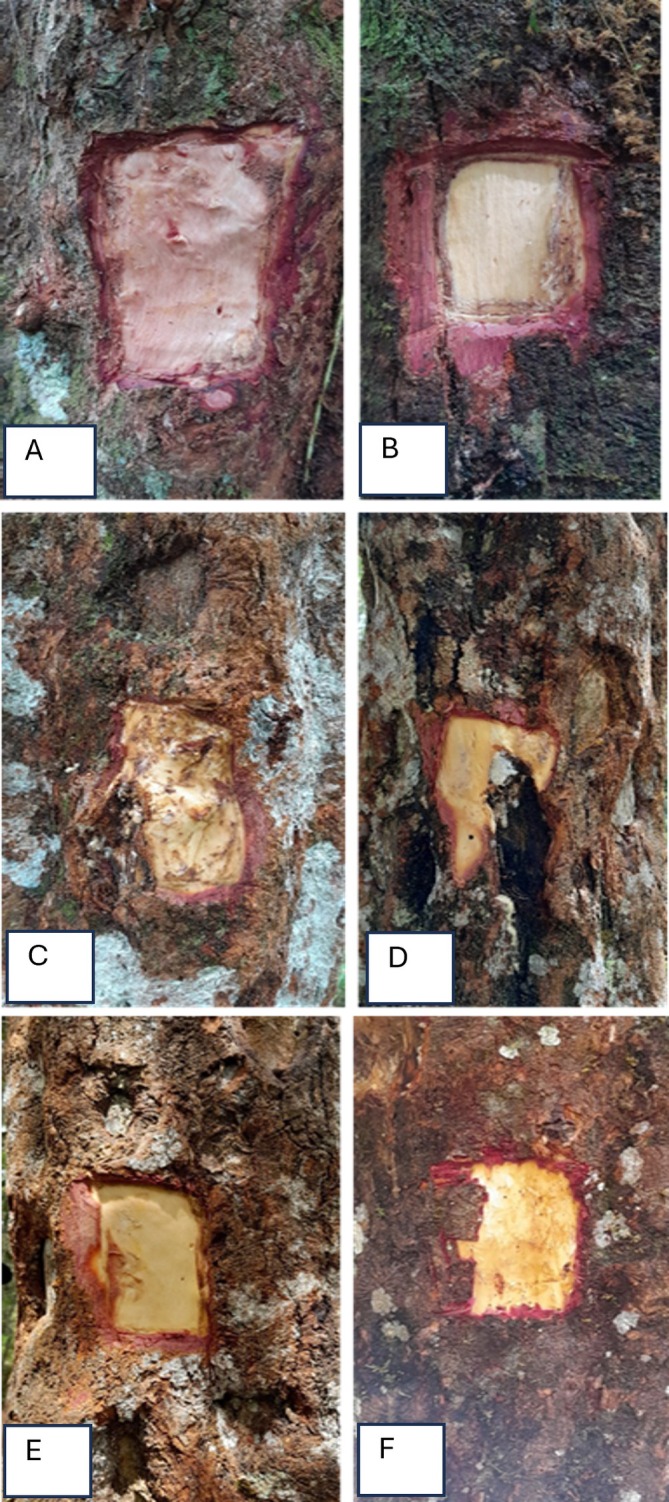
The part of the plant which was sampled. Untapped tree from community forest (A), the untapped tree from the natural forest (B), wounded bark of the tapped tree from community forest (C), wounded bark of the tapped tree from the natural forest (D), unwounded bark of the tapped tree from community forest (E), unwounded bark of the tapped tree from natural forest (F).

Sampling was conducted in natural forests and community forests in the Humbang Hasundutan area, North Sumatra, Indonesia. Samples were taken from styrax trees that met the specified criteria (Figure 1). Samples were coded and separated based on tree origin, tapped and untapped (Table 1). Samples were taken aseptically and carefully by peeling the bark and then storing them in an ice box at a temperature of −20°C. The samples used in this study included three individual trees that had been tapped and those that had not been tapped, with a combined total of 18 samples. The limited sample size of *n* = 3 was selected partly due to (1) the destructive nature of the sampling methodology and (2) conservation considerations to minimise impacts on natural forest populations. This approach aligns with sample sizes employed in comparable studies of endangered tree species (Gillon et al. 2023; Ferreira et al. 2024).

**TABLE 1:** Sampling location.

Code	Treatment		Location	GPS coordinate		
				Tree 1	Tree 2	Tree 3
A	Untapped tree	(Never wounded)	Community forest	2°15′38.9″ N 098°39′24.5″ E	2°15′40.6″ N 098°39′22.1″ E	2°15′41.3″ N 098°39′21.9″ E
B			Natural forest	2°15′42.0″ N 098°39′23.7″ E	2°15′42.2″ N 098°39′23.9″ E	2°15′42.4″ N 098°39′23.9″ E
C	Tapped tree	Wounded bark	Community forest	2°15′40.1″ N 098°39′20.5″ E	2°15′38.8″ N 098°39′20.7″ E	2°15′38.1″ N 098°39′22.2″ E
D			Natural forest	2°15′41.8″ N 098°38′25.2″ E	2°15′41.6″ N 098°39′25.1″ E	2°15′40.9″ N 098°39′24.9″ E
E		Unwounded bark	Community forest	2°15′40.1″N 098°39′20.5″ E	2°15′38.8″N 098°39′20.7″ E	2°15′38.1″ N 098°39′22.2″ E
F			Natural forest	2°15′41.8″ N 098°38′25.2″ E	2°15′41.6″ N 098°39′25.1″ E	2°15′40.9″N 098°39′24.9″ E

This study focused specifically on microbial communities associated with frankincense stems, particularly in the context of tapping‐induced injury, as prior research has demonstrated that stem wounds significantly alter microbial colonisation and resin secretion (El‐Nagerabi et al. 2014). Given that microbial interactions in *Boswellia* species are strongly influenced by stem damage, whether natural or human‐induced (Al‐Harrasi et al. 2019), this investigation excluded other tree regions (e.g., leaves and roots) with primary focus on stem‐specific microbiomes (Khan et al. 2017).

### 2.2 Targeted Amplicon Metagenomic Analysis

#### 2.2.1 Genomic DNA Extraction and Purification

Total genomic DNAs from stem samples were extracted using a combination of the Cetyltrimethylammonium bromide (CTAB) protocol (Li, Wang, et al. 2013) and DNA extraction kit (Quick‐DNAPlant/Seed Miniprep Kit, Zymo Research, D6020). The stem samples (≤ 150 mg each) were ground in liquid nitrogen, added directly to a ZR Bashing Bead lysis tube (2.0 mm) and efficiently lysed by rapid bead‐beating without the use of organic denaturants or proteinases. Polysaccharides and polyphenols/tannins were removed from the DNA using ZymoSpin apparatus containing Zymo‐Spin III‐HRC filter and a PCR inhibitor removal column.

#### 2.2.2 Sequencing

The extracted genomic DNAs were PCR‐amplified using the 16S rRNA genes of distinct regions (16SV3‐V4) and a specific barcoded primer (16S V4: 515F‐806R). PCR reactions were carried out with Phusion High‐Fidelity PCR Master Mix (New England Biolabs). Sequence libraries were generated using NEBNext Ultra DNA Library Pre Kit for Illumina, following the manufacturer's recommendations, and index codes were added. The quality of the PCR products was determined using Qubit 2.0 Fluorometer (Thermo Scientific) and Agilent Bioanalyzer 2100 system. Subsequently, the library was sequenced under the Illumina platform, and 250 bp paired‐end reads were generated in PT. Genetika Science.

#### 2.2.3 Alpha Diversity

Operational Taxonomic Units (OTUs) were considered for the alpha diversity analysis to assess the richness and diversity of microbial communities within individual samples (Li, Zhang, et al. 2013). Boxplots were generated to visualise the differences in alpha diversity indices across groups. The alpha diversity analysis was carried out using Qiime software v. 1.9.1 and further analysed and visualised in RStudio v. 4.0.3. These indices include ACE (Abundance‐based Coverage Estimator) for estimating OTU richness, Chao1 for estimating species richness, OB (Observed species) for directly observed species count, Shannon index for evaluating taxa diversity and evenness (higher values indicating greater community diversity and species evenness), and Simpson index for characterising species diversity and evenness within communities.

#### 2.2.4 Beta Diversity

Beta diversity was assessed using weighted and unweighted UniFrac distance matrices to incorporate phylogenetic relationships among microbial taxa (Lozupone et al. 2011). Principal Coordinates Analysis (PCoA) was then performed for visualisation and interpretation of the community dissimilarities. PCoA reduces the complex distance data into principal coordinate axes, enabling visualisation of the major sources of variation across samples in two or three dimensions. This approach facilitates the identification of clustering patterns and differences in microbial community composition.

#### 2.2.5 Data Analysis

Sequencing was performed on a high‐throughput platform, generating raw data that was processed using Qiime 2 (v2022.8). Denoising and chimera filtering were performed using the DADA2 plugin with truncation at 250 bp and trimming of 10 bp from the 5′ end. Taxonomic classification was done using a Naive Bayes classifier trained on the SILVA 138 database (99% OTUs), with a confidence threshold of 0.7. The bioinformatics pipeline included quality filtering, chimera removal and operational taxonomic unit (OTU) clustering. Taxonomic classification was achieved through comparison with reference databases, and diversity metrics—both alpha (within‐sample diversity) and beta (between‐sample diversity)—were calculated. Statistical analyses and visualisations were used to compare microbial composition and diversity among the groups, highlighting potential correlations with tree tapping, bark wounding and forest type. The statistical analysis for alpha diversity includes significance testing using the Wilcoxon test. This analysis was conducted independently and is not influenced by the nested experimental design, as it focuses solely on group‐level comparisons rather than accounting for hierarchical structure.

## 3 Result and Discussions

### 3.1 Bacteria Diversity and the Difference Between Groups

The result showed that tapped trees exhibited a greater trend of microbial diversity and abundance than untapped trees, even though not statistically significant, largely due to their increased exposure to external microbial communities and environmental elements (Figure S1). When a tree is tapped, it creates an open wound that allows soil, air, water and microbes to enter the exposed tissues. These wounded sections can stimulate specific microbial groups that assist the tree in adapting to environmental stress and bolster its defence mechanisms (Williams et al. 2024). Additionally, tapping initiates the flow of exudates, which can attract various microbes to the tree (Mishra et al. 2022).

Regarding tree ecosystems, natural forests exhibit higher diversity and evenness than community forests, likely due to reduced human interference, such as logging, agriculture and construction, which allows for stable ecosystems (Figure S2). Natural forests, with minimal human interference, exhibit stable ecosystems supported by nutrient‐rich soils and diverse ecological niches, fostering microbial diversity (Sardar et al. 2023; Petr et al. 2016). Conversely, alpha diversity is higher in community forests, likely due to active management practices, diverse plant species and varied soil conditions, which create favourable habitats for microbial communities (Baker et al. 2013; Matei et al. 2020). This higher diversity enhances ecological resilience, promoting ecosystem functions like nutrient cycling and carbon sequestration (Chaer et al. 2009). These insights highlight the value of community forests in biodiversity conservation and ecosystem management.

**FIGURE 2:**
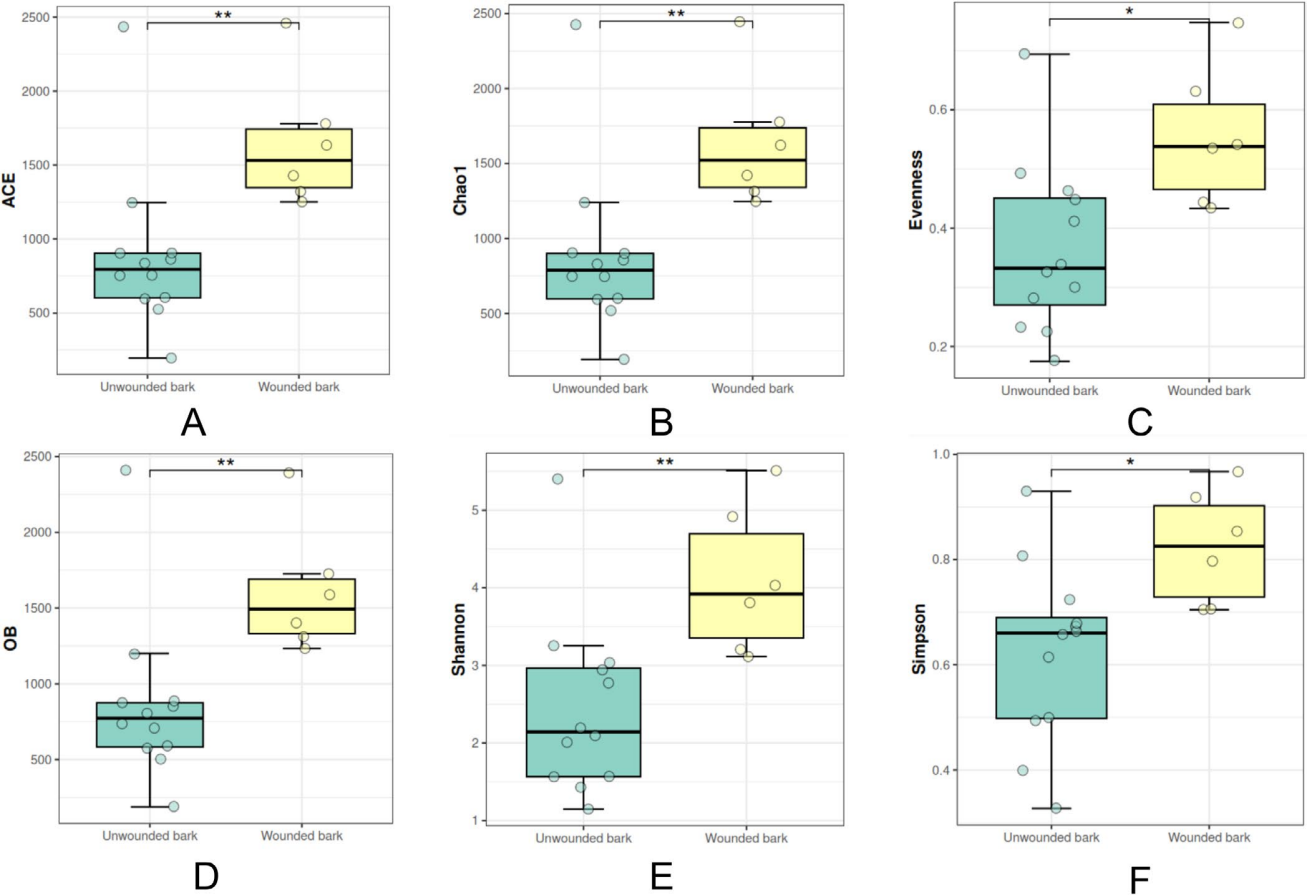
Alpha Diversity Unwounded and Wounded Bark. (A) ACE (Abundance‐based Coverage Estimator): Estimates species richness based on rare species observed in the sample; (B) Chao1: A non‐parametric estimator of species richness, emphasising rare taxa. (C) Evenness: Reflects the relative distribution of species abundances within the community. (D) Observed OTUs (OB): The actual count of operational taxonomic units (OTUs) identified in the samples. (E) Shannon Index: Measures species diversity, accounting for both abundance and evenness of the species present. (F) Simpson Index: Indicates species dominance, with lower values representing higher diversity. *p < 0.05 (significant at the 5% level), **p < 0.01 (significant at the 1% level).

Wounded bark exhibits greater microbial diversity and evenness than unwounded bark (Figure 2), similar to the results observed in tapped versus untapped trees. The exposed area on the wounded bark provides an entry point for various external microbes, which can colonise and establish themselves in the newly accessible tissue (Williams et al. 2024). This increased exposure facilitates a more diverse microbial community, as the open wound invites colonisation by airborne and surface‐contacting microbes (Wysocki 2002). The diversity in these exposed regions reflects how environmental access points allow for greater microbial variety, often limited in protected, intact bark areas (Dreyling et al. 2024).

**FIGURE 3:**
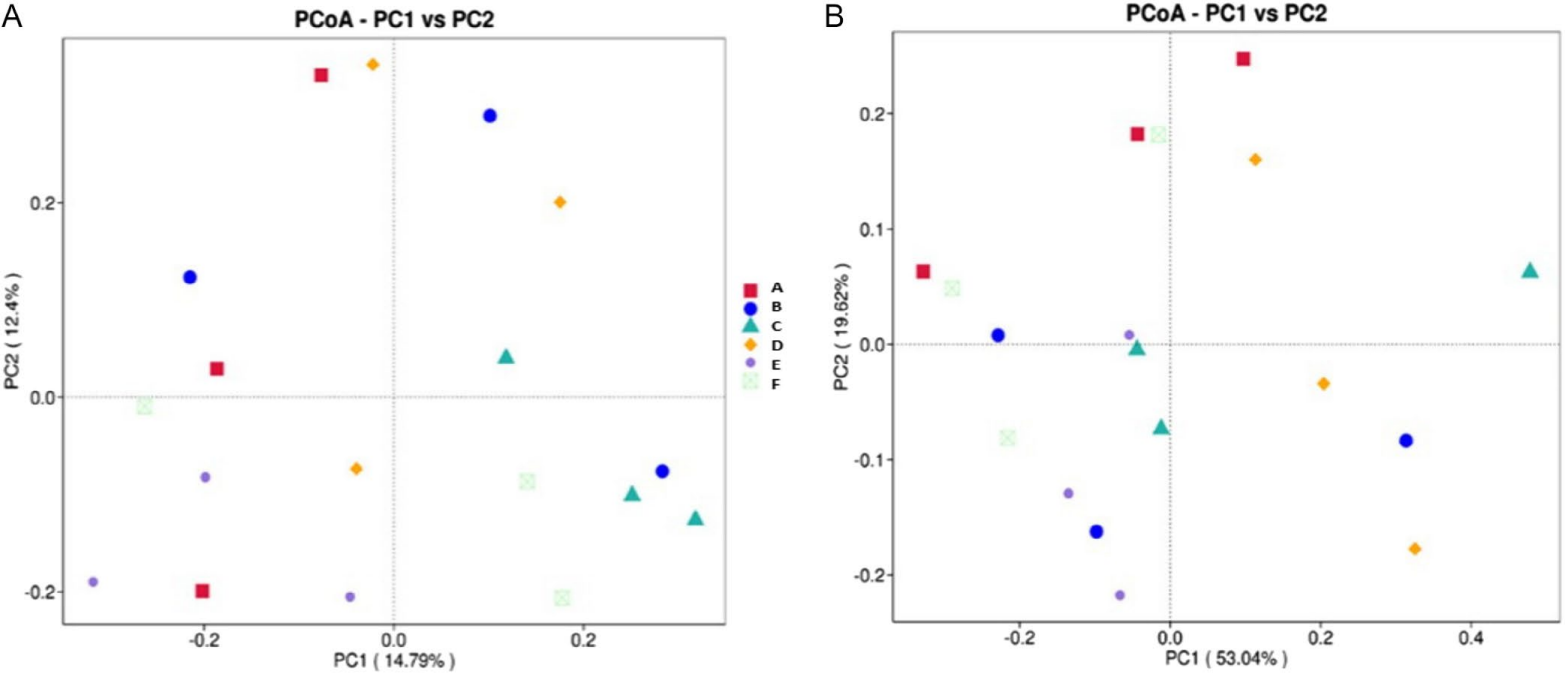
Beta Diversity visualised in Principal Coordinates Analysis (PCoA) (A) Unweighted UniFrac Distance (B) Weighted UniFrac Distance. (A) ACE (Abundance‐based Coverage Estimator): Estimates species richness based on rare species observed in the sample; (B) Chao1: A non‐parametric estimator of species richness, emphasising rare taxa. (C) Evenness: Reflects the relative distribution of species abundances within the community. (D) Observed OTUs (OB): The actual count of operational taxonomic units (OTUs) identified in the samples. (E) Shannon Index: Measures species diversity, accounting for both abundance and evenness of the species present. (F) Simpson Index: Indicates species dominance, with lower values representing higher diversity.

The results of beta diversity represented in principal coordinate analysis (PCoA) using Unweighted and Weighted UniFrac distance can be seen in Figure 3. Principal coordinates analysis (PCoA) based on Unweighted and Weighted UniFrac distances revealed distinct patterns in microbial community structure across sample groups. The Unweighted UniFrac analysis, which considers only the presence or absence of taxa, explained 14.79% and 12.4% of the variation along the first two axes, respectively. Although the proportion of variance captured was relatively low, some group‐level differentiation was observed, with partial clustering of samples such as B and D. This suggests that microbial community varies across different environmental conditions or treatments, although these differences are not pronounced when considering only taxonomic presence (Clark et al. 2021).

In contrast, the Weighted UniFrac plot, which incorporates both phylogenetic information and relative abundances, explained a substantially greater proportion of the variance (PC1: 53.04%, PC2: 10.62%). This indicates that differences in the abundance of shared taxa are a major driver of community dissimilarity. A more apparent separation among groups, particularly between E and F, suggests a strong differentiation in dominant microbial populations. These results imply that environmental factors, such as forest type, bark condition or tapping practices, have a significant influence on microbial community structure at both compositional and abundance levels. Collectively, these findings highlight the importance of using both presence–absence and abundance‐weighted metrics to capture the full extent of microbial diversity and community dynamics in environmental systems (Xia and Sun 2023).

### 3.2 Dominant Taxa

**FIGURE 4:**
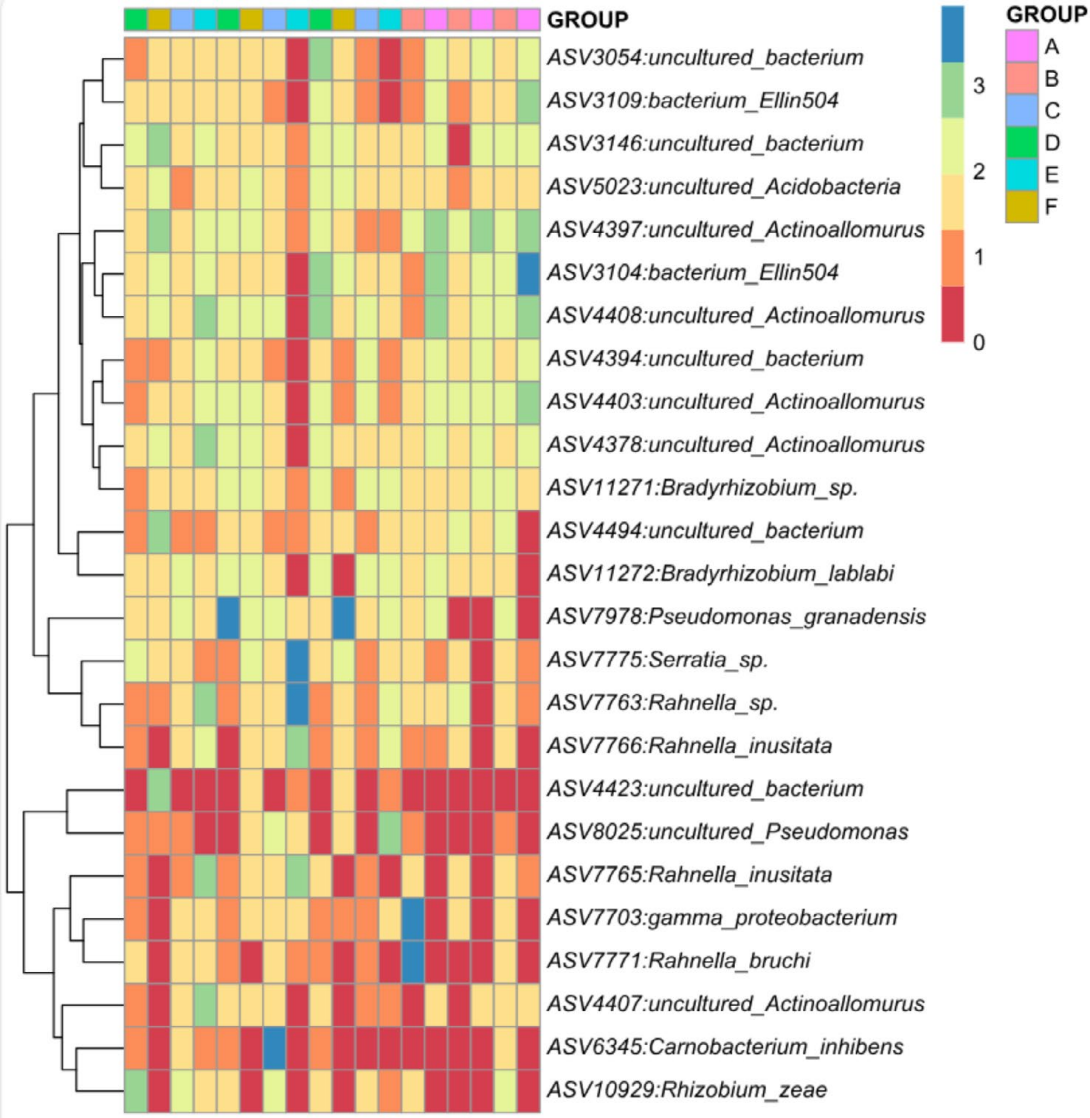
Heatmap of microbial species clusters (A = untapped tree, never wounded, community forest; B = untapped tree, never wounded, natural forest; C = tapped tree, wounded bark, community forest; D = tapped tree, wounded bark, natural forest; E = tapped tree, unwounded bark, community forest; F = tapped tree, unwounded bark, natural forest).

Based on the heatmap of microbial species clusters (Figure 4), it shows that *Bradyrhizobium lablabi* is found abundantly in untapped tree‐never wounded‐community forest (A), tapped tree‐unwounded bark‐community forest (E) and tapped tree‐unwounded bark‐natural forest (F). It is rarely found in untapped tree‐never wounded‐natural forest (B), tapped tree‐wounded bark‐community forest (C) as well as tapped tree‐wounded bark‐natural forest (D). This is believed to be due to a shared characteristic among samples A, E and F: the unwounded bark. The difference in the abundance of *Bradyrhizobium lablabi* between unwounded bark and wounded bark is caused by differences in microhabitat conditions in the two types of bark. Unwounded bark provides a more stable environment and is protected from external disturbances, such as changes in humidity, temperature and exposure to pathogens or competing microbes, thus supporting the growth of more sensitive microbes such as *B. lablabi* . This is similar to the findings of Landsman and Thiel (2021) that habitat characteristics and climatic factors influenced microhabitat, arthropod communities and microbial communities on tree bark. Conversely, wounded bark tends to attract colonisation by other microbes, including pathogens or opportunistic microbes, which are more competitive than *B. lablabi* , thus reducing its abundance. In addition, wounds on tree bark trigger the production of defence metabolites such as phenols and tannins that have antimicrobial properties, which may affect *B. lablabi* more than other microbes. Changes in microhabitat due to wounds, such as increased oxygen exposure or decreased humidity, also create less than ideal conditions for *B. lablabi* (Gauthier et al. 2015; Othman et al. 2019; Raitanen et al. 2020).

*Bradyrhizobium lablabi* is a nitrogen‐fixing bacterium that forms symbiotic associations with leguminous plants, particularly *Lablab purpureus* (hyacinth bean) (Chang et al. 2011). By forming root nodules, *B. lablabi* establishes a mutualistic relationship with its host, supplying fixed nitrogen in exchange for carbon compounds derived from photosynthesis. Additionally, it contributes to soil nitrogen cycling, improves plant productivity and promotes sustainable agriculture, particularly in nutrient‐poor or degraded soils. Its adaptability to various environmental conditions makes it a valuable microbe for ecological restoration and sustainable farming systems (Grönemeyer et al. 2017; Lamrabet et al. 2023). Figure 4 also showed that *Pseudomonas granadensis*, *Serratia* sp. and *Rahnella* sp. are found abundantly in A and are rarely found in E and F. This is probably because sample A was untapped while samples E and F were tapped. The abundance of *Pseudomonas granadensis*, *Serratia* sp. and *Rahnella* sp. in untapped trees and their rarity in tapped trees can be attributed to the stability and undisturbed nature of the microhabitat on untapped bark. Untapped trees maintain more stable environmental conditions such as humidity, temperature and oxygen levels, which are conducive to the growth of these microbes. In contrast, tapping causes physical wounds that alter the bark's structure, increasing oxygen exposure and changing humidity, which can disrupt the stable environment and make it less suitable for these species. Additionally, tapped trees often produce defence compounds like phenols and tannins in response to the wound, which have antimicrobial properties and can suppress the growth of more sensitive microbes like *Pseudomonas granadensis*, *Serratia* sp. and *Rahnella* sp. (Baldrian 2017). In general, *Bradyrhizobium*, *Serratia* and *Pantoea* are bacterial genera with significant roles in promoting plant health and potentially influencing resin production. *Bradyrhizobium* species are renowned for their symbiotic nitrogen fixation capabilities, particularly in legumes, enhancing plant growth and yield. For instance, co‐inoculation with *Bradyrhizobium* and other beneficial microbes has been shown to improve nodulation and nitrogen uptake in crops like mung bean and soybean (da Costa Neto et al. 2024; Win et al. 2023). Additionally, certain *Bradyrhizobium* isolates produce indole‐3‐acetic acid (IAA), a phytohormone that stimulates root development, potentially influencing secondary metabolite pathways involved in resin synthesis (Tiwari et al. 2025; Seidu et al. 2025). *Serratia* species, such as *Serratia marcescens* , exhibit plant growth‐promoting traits including IAA production, phosphate solubilisation and antagonistic activity against plant pathogens (Trinh and Nguyen 2024). These characteristics contribute to enhanced plant growth and resilience against diseases (Fanai et al. 2024). Similarly, *Pantoea* species are recognised for their abilities in nitrogen fixation, phytohormone production and biocontrol activities, which collectively improve plant stress tolerance and disease resistance (Singh et al. 2021; Fanai et al. 2024). The synergistic effects of these microbes can lead to improved plant health and potentially augment resin production through hormonal modulation and enhanced nutrient acquisition.

Furthermore, tapping can lead to increased competition from opportunistic and pathogenic microbes that are better adapted to the disturbed conditions of the wound site. These microbes can exploit the changes in the microenvironment caused by tapping, such as increased nutrient availability or changes in the tree's physiological processes. As a result, the more resilient species outcompete *Pseudomonas granadensis*, *Serratia* sp. and *Rahnella* sp., leading to their reduced abundance in tapped areas. The combination of microbial competition, environmental stress and the tree's defence mechanisms creates conditions that favour other microbial species over those typically found in the more stable and less disturbed environments of untapped trees (Lagacé et al. 2004).

### 3.3 Human Interaction and Its Impact

**FIGURE 5:**
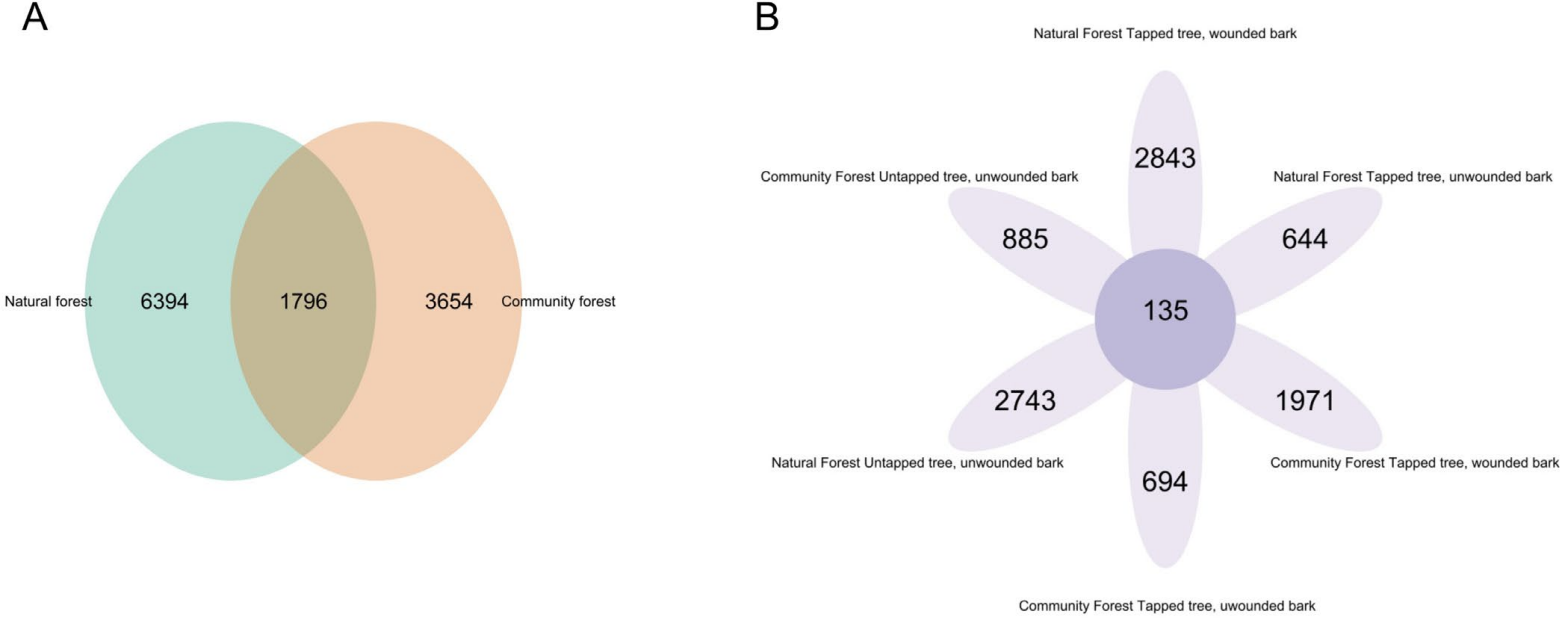
Venn diagram of (A) Natural Forest and community forest and (B) Each sampling treatment of natural and community forest.

According to alpha diversity, human interference did not leave a large impact between different forest types; however, it can be identified that the alpha diversity indices are higher in natural forest than in community forest (Figure S2). This result is in agreement with the Venn diagram (Figure 5). Natural forest has more unique OTUs than community forest (Figure 5A), while according to the tapping and wound information, tapping treatment may increase the microbial abundance (Figure S1, Figure 5B).

**FIGURE 6:**
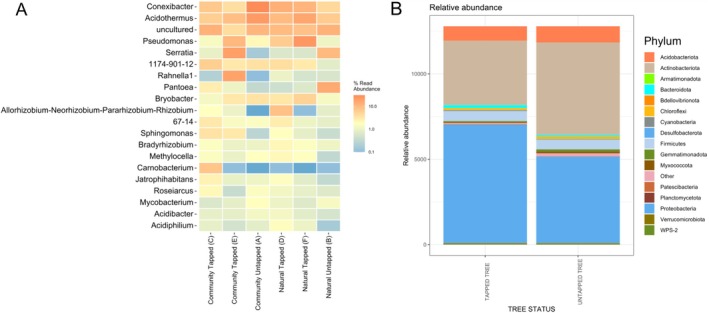
Taxonomic analysis of bacteria from community forest and natural forest. (A) from genus level, (B) from phylum level.

At the genus level, taxonomic analysis (Figure 6A) showed that *Conexibacter* and Acidothermus were abundant across all samples. Interestingly, *Conexibacter* and *Acidothermus* were more prevalent in untapped community forests than in untapped natural forests. In community forests, tapping reduced their abundance, while in natural forests, tapping increased their abundance. This suggests these microbes may respond differently to tapping based on forest type. *Pseudomonas* was more abundant in tapped natural forests than community forests, indicating it may thrive under the conditions introduced by tapping in natural forests. *Serratia*, on the other hand, was more abundant in untapped natural forests but declined with tapping. In community forests, *Serratia* was initially less abundant in untapped trees and further decreased with tapping. These findings suggest that *Serratia* responds differently based on forest type and tapping treatment. These patterns suggest that microbial communities respond variably to tapping, potentially due to differences in environmental conditions, nutrient availability, or tree‐microbe interactions in each forest type. It might be insightful to explore the ecological roles of these genera further, as their responses to tapping could relate to specific functions or adaptations in these environments.

At the phylum level, Figure 5B shows that bacterial composition is largely similar between community and natural forests. Natural forests had slightly higher levels of Proteobacteria and Acidobacteriota, while Actinobacteria was more prevalent in community forests. This could suggest that Proteobacteria and Acidobacteriota are well‐suited to conditions often found in natural forests—Proteobacteria tend to thrive in nutrient‐rich environments (Tang et al. 2021), while Acidobacteriota can adapt to more acidic conditions, which may be more common in natural settings (Kang et al. 2022). This result, along with the study of Kang et al. (2022), showed that Proteobacteria and Acidobacteria were abundant in the soil of the National Nature Reserve. In contrast, the higher presence of Actinobacteria in community forests might reflect adaptations to conditions there, potentially influenced by human management practices like soil treatments or different vegetation, which could favour Actinobacteria growth.

In taxonomic analysis, at genus level, *Serratia* and *Pantoea* are more abundant in untapped natural forest than in untapped community forest (Figure 6A). Natural forest is more abundant in Proteobacteria and Acidobacteriota, while less abundant in Actinobacteriota than community forest (Figure 6B).

The genus *Serratia* is well‐known for decades, especially in the clinical field (Williams et al. 2022). It has been recorded to have great potential in producing bioactive products and biocontrol agents; it can produce β‐lactamantibiotics or the antifungal compound oocydin A (Soenens and Imperial 2020). Some species from this genus have been applied as biocontrol agent for pest damages (Grimont et al. 1988; Hurst et al. 2004; Nunez‐Valdez et al. 2008; Rodríguez‐Segura et al. 2012).

The impacts and mechanisms of *Pantoea*—plant interactions are still not clear so far; species from this genus have been recorded as both pathogen and symbiotroph (Doni et al. 2021; Krawczyk et al. 2020; Xue et al. 2021). The current understanding of its beneficial impacts on plants is growth promotion, phytohormone production, abiotic alleviation, while the pathogenic impacts are disease inhibition and wall degrading enzymes related (Lv et al. 2022).

In this study, the community forest has less abundant *Serratia* and *Pantoea* than the natural forest, which might indicate that human interference decreases forest health and resilience as less potential biocontrol agent and plant promotion bacteria can be found in the forest. This impact might be more severe with years.

## 4 Conclusion

In conclusion, the amplicon metagenomic analysis of microbial diversity in Styrax trees revealed significant differences between tapped and untapped trees, particularly in the abundance and diversity of bacteria. Tapped trees exhibited greater microbial diversity and abundance compared to untapped trees. The tapping process created open wounds that allowed soil, air and water microbes to enter the exposed tissues, stimulating specific microbial groups and increasing microbial variety. These findings underscore the impact of human interference on the microbial composition of Styrax trees and highlight the ecological significance of tapping in promoting microbial diversity. Furthermore, the taxonomic analysis revealed distinct responses of bacterial genera to tapping and forest type. This indicates that microbial communities respond variably to tapping and environmental conditions. The higher microbial diversity in community forests can be attributed to active management, plant diversity, soil conditions and ecological resilience. This further underscores the importance of community forests in promoting biodiversity in forest ecosystems. These insights can inform future conservation and management strategies to enhance biodiversity in forest ecosystems, sustainable forest management practices as well as preserve microbial diversity and ecosystem health. The implications of these findings are significant, as higher microbial diversity in community forests could enhance ecosystem functions such as nutrient cycling, soil health and resilience to environmental stressors.

## Author Contributions

**Arida Susilowati:** funding and project administration, conceptualisation, sampling. **Margaretta Christita:** conceptualisation, sampling, data analysis, writing manuscript. **Siti Halimah Larekeng:** conceptualisation. **Adebola Azeez Lateef:** amplicon metagenomic analysis, bioinformatics work. **Wenzi Ren:** writing manuscript, editing. **Abiodun A. Azeez:** amplicon metagenomic analysis, bioinformatics work. **Fred O. Asiegbu:** funding and project administration, conceptualisation, editing. **Rumella Simarmata:** sample preparation, writing manuscript, editing. **Yeni Khairina:** sample preparation, work on figures and tables. **Fiqriah Hanum Khumairah:** work on figures and tables, writing manuscript. **Deni Elfiati:** funding and project administration.

## Conflicts of Interest

The authors declare no conflicts of interest.

## Supporting information


**Figure S1:** Alpha Diversity Tapped and Untapped Tree. (A) ACE (Abundance‐based Coverage Estimator): Estimates species richness based on rare species observed in the sample; (B) Chao1: A non‐parametric estimator of species richness, emphasising rare taxa. (C) Evenness: Reflects the relative distribution of species abundances within the community. (D) Observed OTUs (OB): The actual count of operational taxonomic units (OTUs) identified in the samples. (E) Shannon Index: Measures species diversity, accounting for both abundance and evenness of the species present. (F) Simpson Index: Indicates species dominance, with lower values representing higher diversity.
**Figure S2:** Alpha Diversity Community Forest and Natural Forest. (A) ACE (Abundance‐based Coverage Estimator): Estimates species richness based on rare species observed in the sample; (B) Chao1: A non‐parametric estimator of species richness, emphasising rare taxa. (C) Evenness: Reflects the relative distribution of species abundances within the community. (D) Observed OTUs (OB): The actual count of operational taxonomic units (OTUs) identified in the samples. (E) Shannon Index: Measures species diversity, accounting for both abundance and evenness of the species present. (F) Simpson Index: Indicates species dominance, with lower values representing higher diversity.

## Data Availability

The data that support the findings of this study are available on request from the corresponding author. The data are not publicly available due to privacy or ethical restrictions.
