# Supplementary material for: Forest Type, Bark Wounding, and Tapping: Their Combined Influence on Bacteria Biota of Styrax Paralleloneurus in Natural and Community Forest
Source: Environ Microbiol Rep. 2025 Sep 4;17(5):e70184. doi: 10.1111/1758-2229.70184 (PMC12411260; doi:10.1111/1758-2229.70184)
Supplement: Supplementary file 1 — Figure S1: Alpha Diversity Tapped and Untapped Tree. (A) ACE (Abundance‐based Coverage Estimator): Estimates species richness based on rare species observed in the sample; (B) Chao1: A non‐parametric estimator of species richness, emphasising rare taxa. (C) Evenness: Reflects the relative distribution of species abundances within the community. (D) Observed OTUs (OB): The actual count of operational taxonomic units (OTUs) identified in the samples. (E) Shannon Index: Measures species diversity, accounting for both abundance and evenness of the species present. (F) Simpson Index: Indicates species dominance, with lower values representing higher diversity. Figure S2: Alpha Diversity Community Forest and Natural Forest. (A) ACE (Abundance‐based Coverage Estimator): Estimates species richness based on rare species observed in the sample; (B) Chao1: A non‐parametric estimator of species richness, emphasising rare taxa. (C) Evenness: Reflects the relative distribution of species abundances within the community. (D) Observed OTUs (OB): The actual count of operational taxonomic units (OTUs) identified in the samples. (E) Shannon Index: Measures species diversity, accounting for both abundance and evenness of the species present. (F) Simpson Index: Indicates species dominance, with lower values representing higher diversity. [file EMI4-17-e70184-s001.docx]

Supplementary Materials


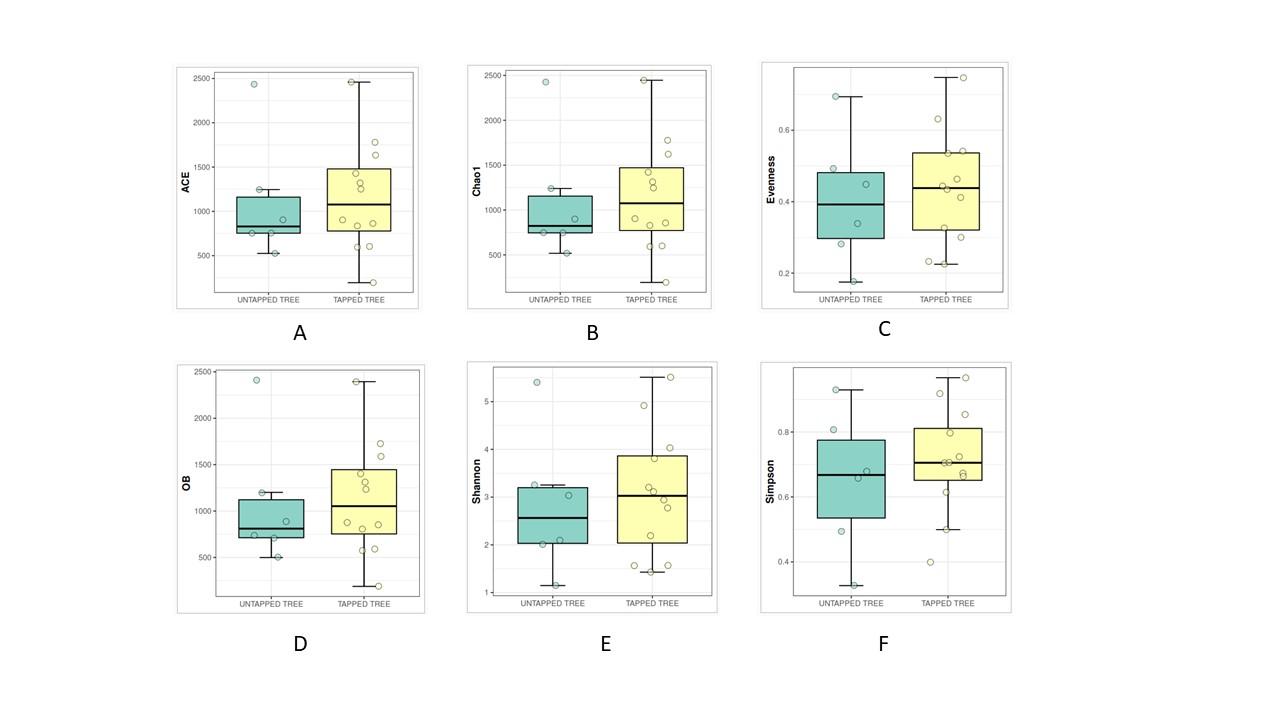


Figure S1. Alpha Diversity Tapped and Untapped Tree. (A) ACE (Abundance-based Coverage Estimator): Estimates species richness based on rare species observed in the sample; (B) Chao1: A non-parametric estimator of species richness, emphasizing rare taxa. (C) Evenness: Reflects the relative distribution of species abundances within the community. (D) Observed OTUs (OB): The actual count of operational taxonomic units (OTUs) identified in the samples. (E) Shannon Index: Measures species diversity, accounting for both abundance and evenness of the species present. (F) Simpson Index: Indicates species dominance, with lower values representing higher diversity.


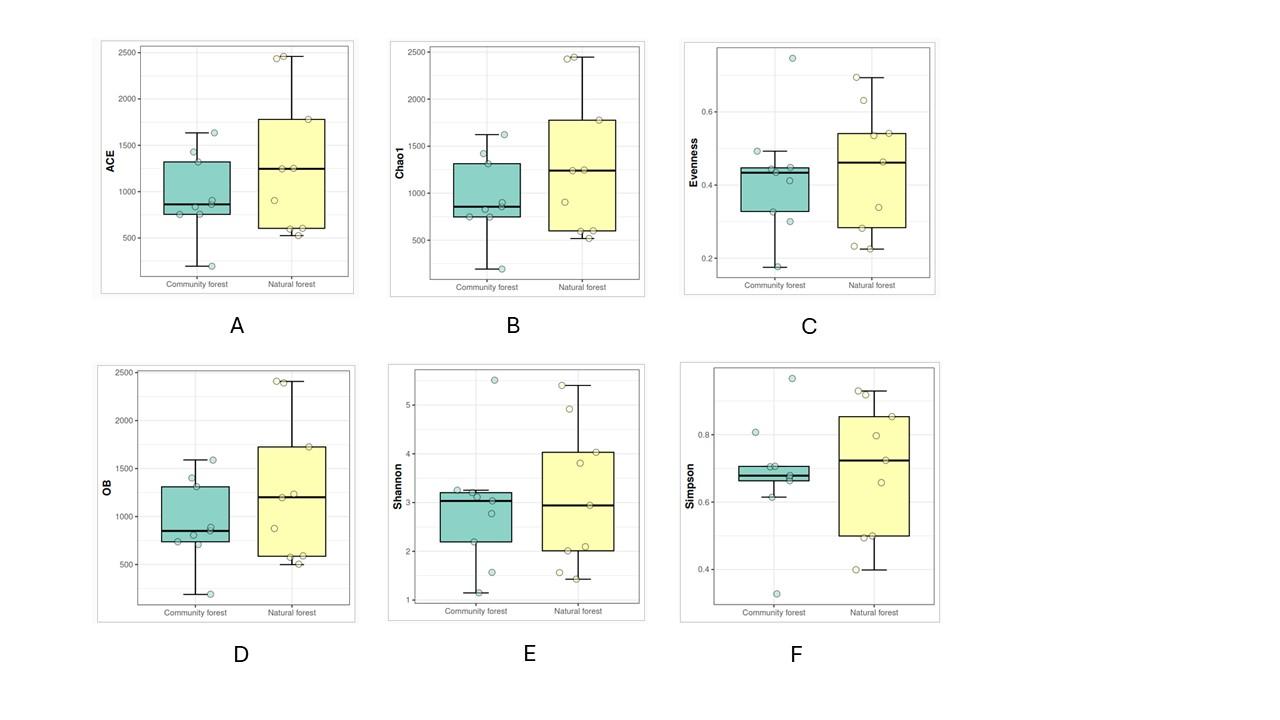


Figure S2. Alpha Diversity Community Forest and Natural Forest. (A) ACE (Abundance-based Coverage Estimator): Estimates species richness based on rare species observed in the sample; (B) Chao1: A non-parametric estimator of species richness, emphasizing rare taxa. (C) Evenness: Reflects the relative distribution of species abundances within the community. (D) Observed OTUs (OB): The actual count of operational taxonomic units (OTUs) identified in the samples. (E) Shannon Index: Measures species diversity, accounting for both abundance and evenness of the species present. (F) Simpson Index: Indicates species dominance, with lower values representing higher diversity.
